# High-Calorie Diet Aggravates Lipopolysaccharide-Induced Pulmonary Inflammation in Juvenile Mice: Association of Acetate-Mediated Macrophage Polarization with AMPK/YAP Signaling

**DOI:** 10.3390/nu18193196

**Published:** 2026-09-27

**Authors:** Futongyu Sun, Hui Liu, Shuxuan Li, Chen Bai, Lina Liu, Yongkuan Ji, Qianqian Li, Xiaohong Gu, Tiegang Liu

**Affiliations:** 1 School of Traditional Chinese Medicine, Beijing University of Chinese Medicine, Beijing 102488, China; 2 Institute of Chinese Medicine Epidemic Disease, Beijing University of Chinese Medicine, Beijing 102488, China; 3 School of Traditional Chinese Medicine, Shandong University of Traditional Chinese Medicine, Jinan 250300, China

**Keywords:** acetate, adenosine 5′-monophosphate (AMP)-activated protein kinase, high-calorie diet, macrophage polarization, pulmonary inflammation, yes-associated protein

## Abstract

Macrophage polarization is an important modulator of the progression, severity, and resolution of pediatric pneumonia. Acetate, a metabolite produced by the gut microbiota, serves as a key regulator of macrophage polarization. Evidence demonstrates that a high-calorie diet (HCD) lowers acetate levels, thereby aggravating pulmonary inflammation, but the underlying mechanism remains unclear. **Objectives:** This study investigated whether HCD-induced exacerbation of pulmonary inflammation in juvenile mice is associated with acetate-mediated macrophage polarization. **Methods:** The pulmonary inflammation model was established in juvenile mice by lipopolysaccharide (LPS) nebulization, with high-calorie diet (HCD) feeding to reduce systemic acetate levels. After model establishment, serum, lung tissue, colon tissue, and feces were collected from each group. Enzyme-linked immunosorbent assay (ELISA), immunohistochemistry (IHC), enzyme-coupled colorimetric assay, Western blotting (WB), and flow cytometry were used to evaluate histopathological changes in the lung and colon, systemic and pulmonary inflammation, acetate levels, and macrophage polarization. Exogenous acetate supplementation was administered to observe its regulatory effect on macrophage polarization, and the downstream signaling pathways were further explored. **Results:** Results revealed that an HCD significantly reduced acetate levels in both lung and fecal samples from juvenile mice with LPS-induced pulmonary inflammation. HCD increased pro-inflammatory cytokines (IL-1β, TNF-α, IL-6) in lung tissue and serum, worsened pulmonary injury, and shifted macrophage polarization toward an M1 phenotype while reducing M2 macrophages. This imbalance was evident in both lung and colon tissues, accompanied by decreased p-AMPK/AMPK and p-YAP/YAP ratios in these tissues. Acetate supplementation and treatment with the AMPK activator Acadesine (AICAR; 5-aminoimidazole-4-carboxamide ribonucleoside) mitigated these effects. **Conclusions:** These findings provide new insights into the association between HCD-induced exacerbation of pulmonary inflammation in juvenile mice and acetate-mediated macrophage polarization, and suggest potential therapeutic strategies. However, given the lack of AMPK inhibition/knockdown and YAP-specific intervention experiments, the involvement of the AMPK/YAP signaling pathway should be interpreted as an association rather than a confirmed causal mechanism.

## 1. Introduction

Pediatric pneumonia is a major acute respiratory infection and remains an important cause of morbidity and mortality in children worldwide [1,2,3,4]. It can be caused by viral, bacterial, or other pathogens and ranges from non-severe to severe disease [3]. The increasing consumption of high-calorie diets (HCDs), characterized by high intake of sugar-sweetened beverages and fast food, has raised concerns regarding their potential impact on respiratory health [5,6]. Poor dietary patterns may aggravate pneumonia by promoting chronic inflammation and impairing host defense against respiratory pathogens [7,8,9]. Clinical studies have shown that overweight and obesity are associated with greater disease severity in children with Mycoplasma pneumoniae pneumonia, with higher BMI correlating with more severe clinical manifestations [10]. Consistently, experimental studies have demonstrated that an HCD and high-fat diet feeding aggravate LPS-induced pulmonary inflammation [11,12,13]. However, the mechanisms linking HCD-associated metabolic alterations to the susceptibility and severity of pediatric pneumonia remain incompletely understood.

Macrophages are key innate immune cells involved in pathogen clearance, antigen presentation, and pulmonary inflammatory responses [14,15]. Alveolar macrophages exhibit substantial plasticity and can adopt distinct functional states in response to environmental signals [16]. Classically activated M1 macrophages, induced by stimuli such as IFN-γ and LPS, produce pro-inflammatory mediators and contribute to host defense, whereas M2 macrophages, induced by IL-4 and IL-13, are primarily involved in inflammation resolution, tissue repair, and immune regulation [17,18]. Dysregulated macrophage polarization has been implicated in excessive pulmonary inflammation and acute lung injury [19]. Thus, factors that regulate macrophage polarization may play an important role in determining the severity of pneumonia.

The gut microbiota is an important regulator of host metabolism and immune function and may influence pulmonary inflammation through the gut–lung axis [20]. Gut microorganisms metabolize dietary components to produce short-chain fatty acids (SCFAs), including acetate, propionate, and butyrate [21,22,23]. SCFAs exert systemic immunomodulatory effects and have been shown to attenuate pulmonary inflammation during respiratory infection and acute lung injury [24,25,26]. Increasing evidence also indicates that SCFAs regulate macrophage function and polarization toward anti-inflammatory phenotypes [27,28,29], potentially through histone deacetylases, G protein-coupled receptors, and other immune signaling pathways [30,31,32,33]. Among these pathways, AMP-activated protein kinase (AMPK) is an important metabolic regulator activated by SCFAs [34] and is involved in macrophage polarization and inflammatory responses [35,36,37]. Reduced AMPK activity may promote YAP activation, which favors pro-inflammatory M1 polarization [38,39,40].

Accumulating evidence indicates that SCFAs influence immune and inflammatory responses in the lungs [41,42]. Our previous studies further demonstrated that a high-calorie diet disrupts the intestinal barrier, reshapes the gut microbiota [12], and markedly decreases SCFA levels in the feces, blood, and lungs of pneumonic mice, with acetate as the major reduced component in lung tissue [43]. Given the established role of AMPK as a downstream effector of SCFAs and the regulatory function of the AMPK/YAP axis in macrophage polarization, we hypothesized that HCD-induced exacerbation of pulmonary inflammation is associated with acetate depletion, reduced AMPK activation, and altered YAP signaling, which may promote M1 macrophage polarization. To test this hypothesis, we established a juvenile mouse model of LPS-induced pulmonary inflammation combined with short-term HCD feeding, and applied acetate supplementation and pharmacological AMPK activation to systematically investigate the role of acetate-regulated macrophage polarization in HCD-aggravated pulmonary inflammation and the underlying mechanisms.

## 2. Materials and Methods

### 2.1. Chemicals and Reagents

Lipopolysaccharide and acetate were purchased from Sigma-Aldrich (St. Louis, MO, USA). AICAR was supplied by MedChemExpress (Monmouth Junction, NJ, USA). Mouse TNF-α, IL-1β and IL-6 ELISA kits were obtained from Elabscience Biotechnology Co., Ltd. (Wuhan, China). Antibodies against AMPK Alpha and YAP1 Polyclonal for immunohistochemistry were sourced from Proteintech Group (Wuhan, China). Antibodies against Phospho-AMPK alpha (Thr172) and Phospho-YAP (Ser127) for immunohistochemistry were acquired from Affinity Biosciences (Chicago, IL, USA). Antibodies against Phospho-AMPK alpha (Thr172), AMPK Alpha and YAP1 Polyclonal for Western blot were sourced from Proteintech Group (Wuhan, China). Antibodies against Phospho-YAP (Ser127) for Western blot and Acetate Assay kit were purchased from Abcam Company (Cambridge, UK). TruStain FcX™ (anti-mouse CD16/32) Antibody, PE anti-mouse CD206 (MMR), PE/Cyanine7 anti-mouse F4/80, APC/Cyanine7 anti-mouse/human CD11b, FITC anti-mouse CD45, Brilliant Violet 421™ Rat IgG2a, κ Isotype Ctrl and PE Rat IgG2a, κ Isotype Ctrl, Brilliant Violet 421™ anti-mouse CD86 for Flow Cytometry were sourced from BioLegend Group (San Diego, CA, USA) Flow cytometry data were analyzed using FlowJo software (BD Biosciences, Franklin Lakes, NJ, USA). Western Blot data were analyzed using ImageJ software (version 1.53t; National Institutes of Health, Bethesda, MD, USA) Statistical analysis was performed using SPSS version 26.0 (IBM Corp., Armonk, NY, USA) and GraphPad Prism version 10.1.2 (GraphPad Software, San Diego, CA, USA).

### 2.2. Animal Experiments

All animal procedures were reviewed. Approval was granted by the Institutional Animal Care and Use Committee of Beijing University of Chinese Medicine. The ethics review numbers were BUCM20250428-003 and BUCM2025021307-1250.

Male SPF-grade C57BL/6 mice, aged four weeks and weighing 15 ± 2 g, were supplied by Beijing Vital River Laboratory Animal Technology Co., Ltd. (Beijing, China). The approval certificate number was SCXK (Beijing) 2021-0006. The animal qualification certificate number was 110011231108478727. The location was Beijing, China. All mice were housed in the animal facility of Beijing University of Chinese Medicine, with 5 mice in each cage. Food access was free. Water access was free. A five-day acclimation period was completed before the experiment. To minimize bias, all experimental mice were assigned unique identification codes by a researcher and underwent stratified randomization based on their initial body weight into five coded groups. Based on previous studies [43] showing a similar effect size, we estimated that *n* = 10 mice per group would be sufficient to detect a statistically significant difference. Throughout the experiment, any animal losses will be documented and reported, with the final *n* for each group clearly indicated in all corresponding figures and tables. No adverse events were reported during the study. To minimize bias, animals were randomly assigned to experimental groups using stratified randomization based on body weight. For indicator measurements, mice were randomly selected from each group, and the number of biological replicates used for each assay is indicated in the figure legends and the corresponding Methods sections. This allocation was designed to avoid using the same tissue for incompatible assays and to ensure sufficient material for each endpoint. Group allocations and corresponding interventions were concealed using a coding system accessible only to the caregivers responsible for daily treatments; all other personnel, including specimen collectors, pathologists, and statisticians, remained blinded until the final analysis. Specimens were collected by blinded staff and labeled with unique identifiers. Independent pathologists evaluated histopathological and immunofluorescence results using only these identifiers, without access to experimental metadata. Statistical analysis was performed by a statistician unaware of group assignments. Although daily caregivers could not be blinded due to obvious phenotypic differences resulting from diet-induced obesity, all endpoint analyses strictly followed predefined blinding protocols. The randomization and grouping methods in Experiment 2 were identical to those used in Experiment 1. The first animal experiment used random grouping. Five groups were established. The groups were the control group, pulmonary inflammation (LPS), HCD, HCD plus pulmonary inflammation (HCD + LPS), and acetate (HCD + LPS + Acetate). The second experiment used the same randomization method. Five groups were included. The groups were the control group, LPS group, HCD + LPS group, HCD + LPS + Acetate group, and HCD + LPS + AICAR group. The maintenance diet was produced by SPF (Beijing, China) Biotechnology Co., Ltd. The HCD was produced by the same company. Quality control followed SPF standards [44]. The HCD provided higher energy density. The value was 1828 kJ per 100 g. The maintenance diet provided 340 kJ per 100 g. The energy level was 5.4 times higher in the HCD. The difference was linked to lipid content. The lipid proportion was 16.10% in the HCD. The lipid proportion was 4.62% in the maintenance diet. The carbohydrate proportion was 58.80% in the HCD. The carbohydrate proportion was 5.25% in the maintenance diet. The protein proportion was 13.73% in the HCD. The protein proportion was 16.26% in the maintenance diet [13,45]. Table 1 presents the design of experiment 1. Mice in the HCD group received the HCD. Mice in the HCD + LPS group received the HCD. Mice in the HCD + LPS + Acetate group received the HCD. Mice in the Control group received the maintenance diet. Mice were fed either the high-calorie diet or the standard maintenance diet for six days. Mice in the LPS group received the maintenance diet. LPS nebulization was performed daily from day 4 through day 6. The concentration was 0.5 mg/mL. Nebulization lasted 30 min. Treatment occurred twice daily. The LPS group received LPS nebulization. The HCD + LPS group received LPS nebulization. The HCD + LPS + Acetate group received LPS nebulization. The Control group received pure water nebulization. The HCD group received pure water nebulization. The volume matched the LPS treatment. The HCD + LPS + Acetate group was given 200 mM sodium acetate solution in drinking water for six days. Table 2 presents experiment 2. The diet plan matched experiment 1. The intervention plan matched experiment 1. The Control group followed the same protocol. The LPS group followed the same protocol. The HCD + LPS group followed the same protocol. The HCD + LPS + Acetate group was given 200 mM sodium acetate in drinking water for six days, but otherwise followed the same protocol as the HCD + LPS group. The HCD + LPS + AICAR group received the same conditions as the HCD + LPS group. AICAR was administered intraperitoneally to this group at a dose of 250 µg/g twice daily for six days, and the remaining groups received an equal volume of PBS by the same route. Administration occurred twice daily. Daily observations were made of general conditions, including coat condition, behavioral patterns, and locomotor activity. Body weight and food intake were also monitored daily. On Day 7, anesthesia was induced. A 2% sodium pentobarbital solution was used. The dose was 0.1 mL per 10 g body weight. Injection was performed by the intraperitoneal route. Blood samples were obtained by cardiac puncture. After blood collection, mice that did not die were euthanized by cervical dislocation, and then colon and lung tissue samples were collected for subsequent analysis. Rinsing of the lung tissues was performed using physiological saline. This step removed residual blood. Colon tissues were rinsed with physiological saline. This step removed feces. This step removed intestinal contents. Tissue surfaces were dried with absorbent paper. The lung index was calculated as (lung wet weight/body weight) × 100%, where the wet weight was measured in grams after removal of excess moisture. For each group, 6 mice were randomly selected for histological, flow cytometric, ELISA, and acetate measurements, and 3 mice were used for Western blotting.

### 2.3. Lung Histopathology

To investigate the effects of an HCD on LPS-induced lung inflammation in young mice, the study conducted pathological analysis of lung tissues using H&E staining as an inflammation marker. Harvested lung tissues were fixed in 4% paraformaldehyde for 24 h. Standard ethanol–xylene dehydration was performed. Paraffin embedding was completed after dehydration. Paraffin blocks were cut in the sagittal plane. Section thickness was set at 5 μm. Tissue sections were prepared for staining. Hematoxylin and eosin staining was applied. Hematoxylin staining lasted 5 min. Eosin staining lasted 3 min. Histological features were examined under an optical microscope. Each experimental group was evaluated separately. Six fields of view were selected at random. Magnification was set at 200×. Images were captured for later analysis.

### 2.4. Colon Histopathology

Colon tissues were fixed in a 4% paraformaldehyde solution for 24 h, followed by dehydration in ethanol–xylene and embedding in paraffin. Sections were cut at 5 μm thickness and stained with H&E (hematoxylin: 5 min; eosin: 3 min) for histological analysis. Six random fields of view at 200 × magnification were selected per experimental group, and photographs were captured for further evaluation.

### 2.5. Enzyme-Linked Immunosorbent Assay (ELISA)

For cytokine measurement, six mice from each group were randomly selected to provide lung tissue and serum samples. The levels of IL-1β, IL-6, and TNF-α in lung tissue and serum were determined using commercial ELISA kits (Elabscience Biotechnology, Wuhan, China) following the manufacturer’s protocols. All samples were analyzed in duplicate, and optical density was measured at 450 nm using a SpectraMax^®^ i3x microplate reader (Molecular Devices, Silicon Valley, CA, USA). Cytokine concentrations were calculated by interpolation from standard curves.

### 2.6. Immunohistochemistry (IHC)

To elucidate the mechanism by which an HCD exacerbates the imbalance of M1/M2 polarization in mice with LPS-induced pulmonary inflammation, this study utilized IHC to examine the expression of AMPK, YAP, *p*-AMPK, and *p*-YAP in mouse lung and colon tissues. For immunohistochemical (IHC) analysis, lung and colon tissues were collected from six randomly selected mice per group for independent biological replicate experiments. Paraffin-embedded sections were dewaxed, rehydrated, and subjected to antigen retrieval by heating in 0.01 M citrate buffer for 20 min, followed by cooling to room temperature and PBS washing. Endogenous peroxidase activity was blocked with 3% H_2_O_2_ for 10 min. Sections were then incubated overnight at 4 °C with primary antibodies against AMPK (1:200), *p*-AMPK (1:100), YAP1 (1:200), and *p*-YAP1 (1:100). After washing, sections were incubated with HRP-conjugated goat anti-rabbit/mouse secondary antibody for 30 min at room temperature, followed by DAB chromogenic detection for 5–10 min. The sections were then dehydrated in graded alcohols, cleared in xylene, and mounted with neutral gum. Positive staining areas were quantified using ImageJ software (version 1.53t; National Institutes of Health, Bethesda, MD, USA).

### 2.7. Fecal/Lung Tissue Acetate Analysis

To further investigate the effect of HCD on acetate levels in lung tissue and feces of LPS-induced young mice, we measured acetate concentrations using an acetate assay kit (Cambridge, UK). For acetate quantification, six fecal samples and six lung tissue samples were randomly selected from each group and measured using an acetate assay kit [46,47]. Both fecal and lung tissue specimens, each weighing 10 mg, were accurately weighed, washed with ice-cold phosphate-buffered saline, and subsequently resuspended in 100 μL of ice-cold Acetate Assay Buffer. The resulting mixture was thoroughly vortexed and centrifuged at 13,000× *g* for 10 min at 4 °C. The supernatant was carefully collected and maintained on ice until further analysis. The acetate concentration was determined according to the formula: Acetate concentration (nmol/mL) = A/B × D, where A is the acetate content (nmol) determined from the standard curve, B is the sample volume added to the reaction well (mL), and D is the dilution factor. The sample was stirred using a horizontal stirrer or pipette, incubated at room temperature for 40 min while avoiding light, and absorbance at 450 nm was measured immediately after removal.

### 2.8. Lung/Colon Tissue Flow Cytometry

To investigate whether the mechanism by which an HCD exacerbates pulmonary inflammation in young mice is linked to an imbalance in macrophage M1/M2 polarization, this study assessed the proportion of M1-like macrophages and M2-like macrophages. Within the CD11b^low^F4/80^+^ macrophage population, M1-like macrophages were defined as CD86^+^CD206^−^ cells and M2-like macrophages as CD86^−^CD206^+^ cells. The M1/M2 ratio was calculated as the proportion of CD86^+^CD206^−^ M1-like macrophages divided by that of CD86^−^CD206^+^ M2-like macrophages. For flow cytometry, lung and colon tissues were collected from six randomly selected mice per group for independent biological replicate experiments. The tissues were dissociated and digested in a solution containing collagenase IV at 37 °C for 30 min. The resulting cell suspensions were filtered through 70-μm cell strainers and enriched by density gradient centrifugation. The cell pellets were collected, resuspended in 1× PBS buffer, and counted, with the cell concentration adjusted to 1 × 10^7^ cells/mL for subsequent use. Aliquots of 100 μL of the cell suspension were transferred to each flow cytometry tube. Then, 1 mL of FVS510 working solution (diluted 1:1000 in PBS) was added to each tube, mixed, and incubated at room temperature in the dark for 15 min. After incubation, 2 mL of PBS containing 1% FBS was added, mixed, and centrifuged at 300× *g* for 5 min, and the supernatant was discarded. Next, 1 μg of TruStain FcX™ (anti-mouse CD16/32, 0.5 mg/mL) [48] was added, mixed, and incubated on ice for 10 min to block Fc receptors. Then, 1 μL each of fluorescently labeled antibodies—CD45 FITC (0.5 mg/mL) [49], CD11b APC-Cy7 (0.2 mg/mL) [49], Brilliant Violet 421™ anti-mouse CD86 (0.2 mg/mL) and F4/80 PE-Cy7 (0.2 mg/mL) [49]—were added to the cell suspension and incubated at room temperature in the dark for 20 min. In parallel, blank control tubes, single-stain compensation tubes, and isotype control tubes were set up for instrument setup, fluorescence compensation, and assessment of nonspecific binding. After incubation, cells were washed with PBS containing 1% FBS, centrifuged at 300× *g* for 5 min, and the supernatant was discarded. The cell pellets were resuspended in fixation buffer and fixed for 15 min at room temperature in the dark. Following fixation, cells were washed twice with permeabilization buffer, centrifuged at 300× *g* for 5 min each time, and then resuspended for intracellular staining. Under light-protected conditions, 1 μL of CD206 PE antibody (0.2 mg/mL) [49] was added, and the corresponding isotype control antibody was added to the isotype control tubes. The mixtures were incubated at room temperature in the dark for 30 min. After incubation, cells were washed once with 1× permeabilization wash buffer, centrifuged at 300× *g* for 5 min, and the supernatant was discarded. The cells were then washed once with 2 mL of PBS containing 1% FBS, centrifuged at 300× *g* for 5 min, and the supernatant was discarded. Finally, the cell pellets were resuspended in 0.5 mL of PBS containing 1% FBS. Data acquisition was performed on a Cytek NL-CLC3000 flow cytometer (Cytek Biosciences, Fremont, CA, USA) using SpectroFlo software, (version 1.0, Cytek Biosciences, Fremont, CA, USA) and data were exported in FCS3.1 format. Flow cytometry data were analyzed using FlowJo software (version 10.8.1, LLC, Ashland, OR, USA). In the FSC vs. SSC scatter plot, the target cell population with a relatively concentrated distribution was gated to exclude cell debris in the lower-left corner. In the FSC-A vs. FSC-H scatter plot, single cells distributed toward the FSC-H axis were gated to exclude doublets. In the FSC-A vs. FVS510 scatter plot, FVS510-negative cells were gated to exclude dead cells. Subsequently, CD45^+^ immune cells were gated, and CD11b^low^F4/80^+^ tissue-infiltrating macrophages were analyzed. The proportions of CD86^+^ M1 macrophages and CD206^+^ M2 macrophages were then determined.

### 2.9. Western blot Analysis

For Western blot analysis, lung and colon tissues were collected from three randomly selected mice per group for independent biological replicate experiments. Lung or colon tissues from each group were thawed, lysed, and homogenized in RIPA lysis buffer. The BCA assay was employed for protein quantification [50]. After SDS-PAGE separation, equal protein loads were electrotransferred onto PVDF membranes. Background was minimized by blocking with 5% skim milk for 2 h at room temperature. The membranes were then incubated overnight at 4 °C with primary antibodies: AMPK (1:10,000), p-AMPK (1:3000), YAP (1:5000), p-YAP (1:10,000), and β-actin (1:10,000). After five washes with TBST (5 min each), the membranes were exposed to HRP-conjugated goat anti-mouse and anti-rabbit secondary antibodies (1:10,000) for 2 h at room temperature. Following further washes, an ECL reaction was performed to acquire images. Target protein expression was quantified by analyzing grayscale values using Image Pro Plus 7.0 software.

### 2.10. Drug Administration

Animal experiments were performed using 10 mice per group for independent biological replicate experiments. AICAR was dissolved in PBS (1×) to prepare a 25 μg/μL solution. The AICAR group, receiving the same diet and nebulized solution as the HCD + LPS group, was administered 250 μg/g of the solution intraperitoneally twice a day. In the HCD + LPS + Acetate group, mice were provided 200 mM sodium acetate solution in drinking water, formulated to match the solution used by other groups, consistent with our previous study [13].

### 2.11. Statistical Analysis

Statistical analysis was performed using SPSS version 26.0 and GraphPad Prism version 10.1.2. Graphical visualization was completed using GraphPad Prism version 10.1.2. The normality of data distribution was assessed using the Shapiro–Wilk test. Homogeneity of variance was evaluated using Levene’s test. Normally distributed continuous data are expressed as the mean ± SD and were analyzed using one-way ANOVA. For comparisons involving multiple groups, Welch’s ANOVA was used when the assumption of homogeneity of variance was violated, followed by the Games–Howell post hoc test for multiple comparisons. When data did not meet the assumption of normality, non-parametric tests were used: the Kruskal–Wallis test followed by Dunn’s post hoc test with Bonferroni correction for multiple comparisons. For longitudinal body weight data, a linear mixed-effects model was used, with group, time, and their interaction as fixed effects and animal as a random effect to account for repeated measurements within the same animal, and multiple comparisons were corrected using Tukey’s test. A *p*-value below 0.05 was considered statistically significant.

## 3. Results

### 3.1. The Mechanism by Which HCD Exacerbates LPS-Induced Pulmonary Inflammation in Mice May Be Associated with Acetate

Throughout the experiment, we monitored changes in body weight among the five mouse groups. No significant differences in body weight were found prior to Day 4. However, starting on Day 5, the HCD + LPS group showed slower weight gain compared to the Control group. On Day 6, body weights in both the LPS and HCD + LPS groups were significantly lower than those in the Control group (*p* < 0.05), whereas the HCD + LPS + Acetate group did not differ significantly from the Control group, suggesting that acetate supplementation may attenuate HCD + LPS-induced body weight loss. (Figure 1A). Histopathological examination of lung tissue revealed that after LPS induction, mice in the LPS group exhibited thickening of alveolar walls and inflammatory cell infiltration around the lung interstitium and bronchial blood vessels. The HCD + LPS group displayed more severe lung damage, with extensive inflammatory cell infiltration and destruction of alveolar structure (Figure 1C). H&E staining of colon tissue revealed that the HCD + LPS group exhibited thinning of the mucosal layer, a reduction in goblet cells, localized congestion, edema, fibrosis in the submucosal lamina propria, and significant crypt loss, compared to the LPS group (Figure S1). Assessment of inflammatory profiles further demonstrated that LPS induction significantly boosted the expression of pro-inflammatory cytokines, specifically IL-1β, IL-6, and TNF-α (Figure 1D,E). Compared with the Control group, the LPS group exhibited significantly elevated levels of these pro-inflammatory factors in both lung tissue and serum. Furthermore, compared with the LPS group, the HCD + LPS group showed significantly higher levels of these pro-inflammatory cytokines in both lung tissue and serum (Figure 1D,E). The results showed that compared with the Control group, the LPS group had significantly reduced acetate levels in both lung tissue and feces (Figure 1F). Compared with the LPS group, the HCD + LPS group exhibited a further significant decrease in acetate levels in both lung tissue and feces (Figure 1F). To explore the intervention effect of acetate, we performed H&E staining and pathological analysis on the HCD + LPS + Acetate group. The results showed that compared with the HCD + LPS group, mice in the acetate supplementation group had thinner alveolar walls, clearer alveolar structures, reduced fusion and rupture, and less inflammatory damage (Figure 1C). In addition, weight loss was more gradual in the HCD + LPS + Acetate group compared with both the LPS and HCD + LPS groups (Figure 1G), and the concentrations of pro-inflammatory cytokines were markedly reduced compared with the HCD + LPS group (Figure 1E–J).

### 3.2. HCD-Induced Exacerbation of Pulmonary Inflammation in Mice Is Associated with M1/M2 Macrophage Polarization Imbalance and with Acetate-Related Effects

Compared to the Control group, the M1/M2 macrophage ratio in the lung tissue of the LPS group was significantly increased (Figure 2C). Similarly, the M1/M2 ratio in the lung tissue of the HCD + LPS group was significantly higher than that of the LPS group (Figure 2C). In contrast, the M1/M2 ratio in the lung tissue of the HCD + LPS + Acetate group was significantly lower than that of the HCD + LPS group (Figure 2C). A similar pattern was observed in the colon: the M1/M2 macrophage ratio in the colonic lamina propria of the LPS group was significantly higher compared to the Control group (Figure 2D). The ratio in the HCD + LPS group was also significantly higher compared to the LPS group (Figure 2D). However, the M1/M2 ratio in the HCD + LPS + Acetate group was significantly lower than in the HCD + LPS group (Figure 2D).

### 3.3. The Exacerbation of M1/M2 Macrophage Polarization Imbalance by HCD May Be Associated with Alterations in the AMPK/YAP Pathway

The results indicated that, compared to the Control group, the LPS group exhibited a significant decrease in the *p*-AMPK/AMPK ratio in both lung and colon tissues (Figure 3B,E and Figure 4B,E). Additionally, the LPS group demonstrated a significant decrease in the *p*-YAP/YAP ratio in both lung (Figure 3C,F) and colon tissues (Figure 4C,F) compared to the Control group. Compared with the HCD + LPS group, the LPS group showed a significant increase in the *p*-AMPK/AMPK ratio in both lung and colon tissues (Figure 3B,E and Figure 4B,E) and a significant increase in the *p*-YAP/YAP ratio in lung (Figure 3C,F) and colon tissues (Figure 4C,F). Conversely, compared to the HCD + LPS group, the HCD + LPS + Acetate group exhibited a significant increase in the *p*-AMPK/AMPK ratio and a significant increase in the *p*-YAP/YAP ratio in both lung and colon tissues (Figure 3B,C,E,F and Figure 4B,C,E,F).

### 3.4. Supplementation with Acetate and Activation of AMPK Were Associated with Mitigation of LPS- and HCD-Induced Pulmonary Inflammation

Pathological examination of lung tissue revealed that after LPS induction, the LPS group exhibited thickened alveolar walls and inflammatory cell infiltration in the pulmonary interstitium, around pulmonary veins, and bronchi. The HCD + LPS group demonstrated more severe lung injury, with pronounced inflammatory cell infiltration and destruction of alveolar structures (Figure 5A). In contrast, both the HCD + LPS + Acetate and HCD + LPS + AICAR groups showed thinner alveolar walls, clearer alveolar structures, reduced alveolar fusion and rupture, and overall alleviated inflammatory damage compared to the HCD + LPS group (Figure 5A). Histopathological analysis of colon tissue sections indicated that the HCD + LPS group exhibited thinning of the mucosal layer, a reduction in goblet cells, and localized congestion, edema, fibrosis in the submucosal lamina propria, and significant crypt loss (Figure S4). However, both the HCD + LPS + Acetate and HCD + LPS + AICAR groups showed less colonic damage compared to the HCD + LPS group (Figure S4). In Experiment 2, the statistical results for body weight showed no significant differences among groups from Day1 to Day4 (*p* > 0.05). On Day5, body weights in the HCD + LPS, HCD + LPS + Acetate, and HCD + LPS + AICAR groups were all significantly lower than those in the Control group (*p* < 0.01). On Day6, body weights in the HCD + LPS, HCD + LPS + Acetate, and HCD + LPS + AICAR groups remained significantly lower than those in the Control group (*p* < 0.01), and body weight in the HCD + LPS group was significantly lower than that in the LPS group (*p* < 0.05) (Figure 5B). The lung index observed in the Control, LPS, HCD + LPS, and HCD + LPS + Acetate groups was consistent with those in Experiment 1, while the HCD + LPS + AICAR group exhibited a trend similar to that of the HCD + LPS + Acetate group (Figure 5C). Additionally, the study measured levels of inflammatory cytokines and found significant increases in pro-inflammatory factors, including IL-1β, IL-6, and TNF-α, following LPS induction (Figure 5D,E). Compared to the LPS group, the HCD + LPS group exhibited further increases in these pro-inflammatory factors in both lung and colon tissues (Figure 5D,E). In contrast, the levels of pro-inflammatory factors in the HCD + LPS + Acetate and HCD + LPS + AICAR groups were significantly lower compared to the HCD + LPS group (Figure 5D,E). Acetate levels in lung tissue and fecal samples were analyzed using an acetate assay kit. Acetate levels in both lung tissues and feces in the HCD + LPS group were significantly lower than those in the LPS group (Figure 5F,G). In contrast, acetate levels in the HCD + LPS + AICAR and HCD + LPS + Acetate groups were significantly higher than those in the HCD + LPS group (Figure 5F,G). These results suggest that both acetate supplementation and AMPK activation can mitigate the exacerbated inflammatory lung injury induced by LPS and HCD. These results suggest that the exacerbation of pulmonary inflammation by HCD may be associated with acetate and the AMPK/YAP signaling pathway. The results shown in Figure 5A–G were derived from independent experimental cohorts using the same animal model as described in Figure 1.

### 3.5. Supplementation with Acetate and Activation of AMPK Attenuated the M1/M2 Macrophage Polarization Imbalance

The data revealed that compared with the Control group, the M1/M2 macrophage ratio was significantly higher in the LPS group (Figure 6B,D). Additionally, the HCD + LPS group exhibited a higher M1/M2 ratio in both the lung and colon compared with the LPS group (Figure 6B,D). In contrast, compared to the HCD + LPS group, both the HCD + LPS + Acetate and HCD + LPS + AICAR groups showed a decrease in the proportion of M1 macrophages and an increase in the proportion of M2 macrophages in both the lung and colon (Figure 6B,D).

### 3.6. The HCD-Induced Exacerbation of LPS-Induced Pulmonary Inflammation May Be Associated with Alterations in the AMPK/YAP Pathway

Consistent with the results of the preceding experiments, compared with the Control group, the phosphorylation of AMPK and YAP was significantly inhibited in both the LPS group and the HCD + LPS group, with the inhibition being more pronounced in the HCD + LPS group (Figure 7A–F, Figure 8A–F). Importantly, compared with the HCD + LPS group, the HCD + LPS + Acetate group and the HCD + LPS + AICAR group exhibited significantly increased ratios of *p*-AMPK/AMPK and *p*-YAP/YAP in both lung and colon tissue (Figure 7A–F, Figure 8A–F). These results suggest that HCD-induced exacerbation of pulmonary inflammation may be associated with reduced acetate levels, decreased AMPK phosphorylation, altered YAP phosphorylation, and aggravated M1/M2 macrophage polarization imbalance. The effects of acetate supplementation were consistent with those of the AMPK activator AICAR.

## 4. Discussion

Pneumonia poses a serious health risk to children worldwide, with high case numbers and mortality rates [51]. Due to their immature immune systems, children are more susceptible to severe illness and even death from pneumonia [52]. In the past decade, accumulating evidence has established a link between the microbiota of the respiratory and intestinal tracts and the pathogenesis of respiratory tract infections [53]. Studies have found that beneficial bacteria in the infant oral cavity, such as *Neisseria*, *Rothia*, and *Prevotella*, are relatively reduced during respiratory tract infections, while harmful bacteria such as *Streptococcus* increase [54]. Hong H et al. further found that upper respiratory tract microbiome diversity is inversely correlated with the severity and length of hospitalization for pneumonia [55]. More importantly, compared with healthy children, children with pneumonia exhibit significant gut microbiota dysbiosis, with decreased abundance of beneficial bacteria and increased abundance of harmful bacteria, confirming that gut microbiota imbalance contributes to lung disease [56]. In summary, the dysbiosis of both respiratory and gut microbiota not only accompanies the onset and progression of pediatric pneumonia but is also closely associated with disease severity and hospital outcomes, suggesting that microbial ecological balance may become a new therapeutic target for the prevention and treatment of pediatric pneumonia.

Accumulating evidence highlights the impact of gut microbiota-derived metabolites on health [57]. SCFAs, key microbial metabolites, are vital for maintaining pulmonary immune homeostasis in children [51], and their balance is influenced by diet [58]. Previous studies show that HCD reduces SCFAs, especially acetate, in feces and serum of LPS-induced pneumonic mice, worsening lung inflammation [13,43]. Acetate also alleviates influenza A virus lung infection [26], yet the mechanism by which HCD aggravates LPS-induced pulmonary inflammation via acetate depletion remains unclear. Here, we used LPS nebulization to induce pulmonary inflammation [59] and a short-term HCD model reflecting children’s unhealthy diets [13,43,45]. Our results show that HCD depletes acetate, exacerbates macrophage polarization imbalance, and intensifies LPS-induced pulmonary injury, and these effects are associated with acetate-mediated modulation of the AMPK/YAP axis.

Growing evidence links various pulmonary diseases to gut microbiota dysbiosis [60]. Microbial metabolites, particularly SCFAs, modulate lung immunity through the gut-lung axis [61]. Despite low pulmonary SCFA concentrations, gut-derived propionate promotes the generation of macrophages from bone marrow precursors, which migrate to the lungs and alleviate allergic inflammation by enhancing IL-10 production [62]. Additionally, acetate enhances the ability of alveolar macrophages to kill Streptococcus pneumoniae [63]. Recent research highlights that AMPK plays a critical role at the intersection of macrophage metabolism and inflammation. AMPK not only governs the metabolic programming of macrophages but also counteracts their inflammatory function, promoting polarization towards an anti-inflammatory phenotype [36]. To explore the potential underlying mechanism, we focused our investigation on this pathway. AMPK, a highly conserved serine/threonine kinase, is considered a key regulator at the interface of nutrient sensing, metabolic flux, and energy homeostasis [64]. When cellular energy levels decline, AMPK is activated. It restores energy balance by promoting ATP production through catabolic pathways, such as glucose uptake and fatty acid oxidation, while inhibiting anabolic processes like gluconeogenesis and lipid synthesis [65]. SCFAs, the end products of anaerobic gut microbial fermentation of dietary fiber, exert multiple beneficial effects on mammalian energy metabolism [66]. SCFAs serve as endogenous ligands for the GPCRs FFAR2 (GPR43) and FFAR3 (GPR41) [67,68], stimulating the secretion of glucagon-like peptide-1 [69,70], which in turn activates downstream signaling cascades such as cAMP-PKA, leading to AMPK phosphorylation [71,72]. Acetate has been shown to activate AMPK and suppress the release of pro-inflammatory cytokines [73,74]. Short-term HCD suppresses AMPK phosphorylation in both lung and colon tissues, intensifying M1/M2 macrophage polarization imbalance. In contrast, acetate supplementation was associated with AMPK activation in both lung and colon tissues and with restoration of M1/M2 polarization equilibrium. Furthermore, intraperitoneal administration of AICAR to mice on an HCD with pulmonary inflammation activated AMPK and alleviated pulmonary inflammation severity, which is consistent with a potential role for acetate in alleviating pulmonary inflammation by activating AMPK in both lung and colon tissues.

High glucose suppresses AMPK activity, leading to the activation of YAP [38], while AMPK maintains energy homeostasis by negatively regulating YAP [40]. YAP, a key transcriptional coactivator in the Hippo pathway, plays critical roles in cell proliferation, immunity, and tissue homeostasis. In macrophages, YAP activation promotes polarization toward the pro-inflammatory M1 phenotype and exacerbates inflammatory responses [75]. This study identifies YAP as a key downstream effector of AMPK, offering new insights into how acetate regulates macrophage polarization. Our study suggests that the HCD-induced reduction in acetate levels and the associated macrophage polarization imbalance may not be confined to the HIF-1α- glycolysis axis [43]; the AMPK/YAP axis may also be involved. We previously showed that the glycolysis inhibitor 2-DG alleviates high-calorie diet-induced lung injury, and the present study demonstrates that the AMPK activator AICAR is equally effective. This comparison suggests that concurrent targeting of both pathways may yield additive protective effects, which is a hypothesis warranting further investigation.

In summary, our research demonstrates that an HCD depletes acetate derived from the gut, exacerbating pulmonary inflammation. This process is associated with acetate-mediated macrophage polarization and the AMPK/YAP pathway. This finding carries important clinical implications for pulmonary inflammation in juvenile mice. The intestinal barrier and a healthy microbiota are critical determinants of healthy maturation in children [39]. Specifically, increasing dietary fiber intake or direct acetate supplementation could serve as adjunctive therapeutic strategies to reduce pulmonary inflammation, particularly in children with pre-existing poor dietary habits. Given the favorable safety profile and low cost of SCFA-based interventions, this approach holds promise for integration into supportive care protocols for pediatric pneumonia. However, the optimal dosing, route of administration, and timing of acetate supplementation in pediatric populations require careful evaluation in future clinical studies. Nevertheless, our results provide a rationale for considering gut microbiota-targeted or metabolite-based nutritional strategies as complementary approaches to standard supportive therapies in pulmonary inflammation.

This study has several limitations. First, the HCD model was based on previous dietary survey data from Chinese children [7], and its nutritional composition has not been thoroughly characterized. Future studies should investigate the interplay between specific dietary components and total caloric intake. Second, the M1/M2 macrophage dichotomy used here represents a simplified model for functional investigation. It is important to note that this classification system was initially defined based on in vitro stimulation conditions, and emerging evidence suggests that it oversimplifies the high plasticity and heterogeneity of macrophage populations in vivo [76,77,78,79]. Future studies should explore the dynamic regulatory effects of HCD on the diverse macrophage phenotypes. Third, due to the similarities between mice and humans in disease development and progression, as well as their high biological and genetic homology, mice are frequently selected as surrogate models for research on disease prevention, drug development, and vaccine evaluation [79]. In this study, we established a pulmonary inflammation mouse model through lipopolysaccharide induction combined with a high-calorie diet. Although this model can mimic certain inflammatory features of pediatric pneumonia, it cannot fully recapitulate the complex etiological background of human disease. Moreover, marked differences persist between mice and humans in pulmonary anatomical structure, airway responsiveness, and the temporal sequence of immune system maturation, which to some extent constrain the clinical extrapolation of our findings. Therefore, caution should be exercised when directly extrapolating these preclinical observations to pediatric pneumonia in humans, and future studies should integrate human cohort data, patient-derived samples, or organoid models to further enhance the translational value of our research findings. Fourth, while our data suggest that acetate exerts its protective effects in association with AMPK activation, we acknowledge that causal evidence is currently lacking. Future studies employing pharmacological AMPK inhibition or genetic AMPK knockdown/knockout in macrophage-specific lineages are warranted to definitively establish that acetate’s effects are causally mediated through AMPK. Such experiments would further strengthen the mechanistic insights of the present study. Fifth, it has been reported in the literature that male mice generally exhibit higher disease susceptibility and more severe pulmonary inflammatory responses in pneumonia models [80]; moreover, estrogen is known to modulate immune cell activity and energy metabolism through the AMPK pathway [81]. Given that the objective of this study is to investigate the potential involvement of the AMPK/YAP axis in pulmonary inflammation-associated lung injury, we chose male mice to establish the disease model in order to minimize potential confounding factors introduced by estrogen, thereby allowing a more focused mechanistic analysis of the pathway itself. At the same time, we fully recognize that the conclusions of this study may not be directly generalizable to females, and we acknowledge this as a clear limitation of the current work.

## 5. Conclusions

The mechanism by which an HCD exacerbates LPS-induced lung injury in mice appears to involve a reduction in acetate levels, which was accompanied by decreased AMPK and YAP phosphorylation, and ultimately aggravates M1/M2 macrophage polarization imbalance (Figure 9). This pathway not only elucidates a nutrient-sensitive inflammatory mechanism in pulmonary inflammation but also provides support for the gut-lung axis, emphasizing how gut-derived metabolites can directly influence pulmonary macrophage function and injury progression.

## Figures and Tables

**Figure 1:**
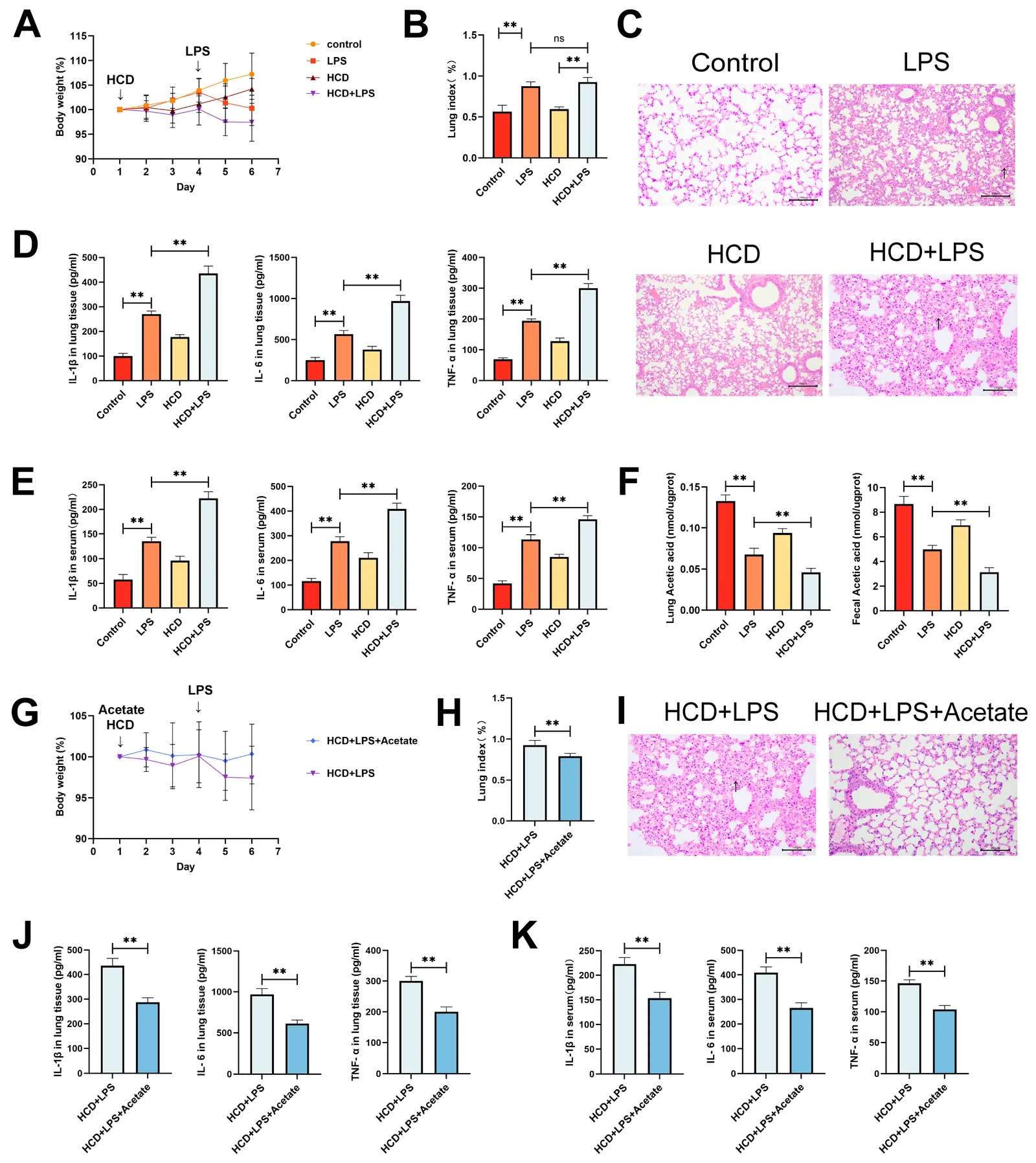
The mechanism by which an HCD exacerbates LPS-induced pulmonary inflammation in mice may be related to acetate. (**A**) Relative body weight changes during the experimental period; Results are presented as mean ± SD, with *n* = 10 mice per group for independent biological replicate experiments. (**B**) Lung index. Results are presented as mean ± SD, with *n* = 10 mice per group for independent biological replicate experiments. (**C**) Lung H&E staining results (200×). The black arrow indicates areas of inflammatory cell infiltration and alveolar wall thickening. (**D**,**E**) ELISA determination of pro-inflammatory cytokines (IL-6, IL-1β, TNF-α) in lung and serum samples from each group; Results are presented as mean ± SD, with *n* = 6 mice per group for independent biological replicate experiments. (**F**) Acetate content in lung and fecal samples from each group, measured by an acetate assay kit. Results are presented as mean ± SD, with *n* = 6 mice per group for independent biological replicate experiments. (**G**) Relative body weight changes during the experimental period; Results are presented as mean ± SD, with *n* = 10 mice per group for independent biological replicate experiments. (**H**) Lung index. Results are presented as mean ± SD, with *n* = 10 mice per group for independent biological replicate experiments. (**I**) Lung H&E staining results (200×). The black arrow indicates areas of inflammatory cell infiltration and alveolar wall thickening. (**J**,**K**) ELISA determination of pro-inflammatory cytokines (IL-6, IL-1β, TNF-α) in lung and serum samples from each group; Results are presented as mean ± SD, with *n* = 6 mice per group for independent biological replicate experiments. ** *p* < 0.01.

**Figure 2:**
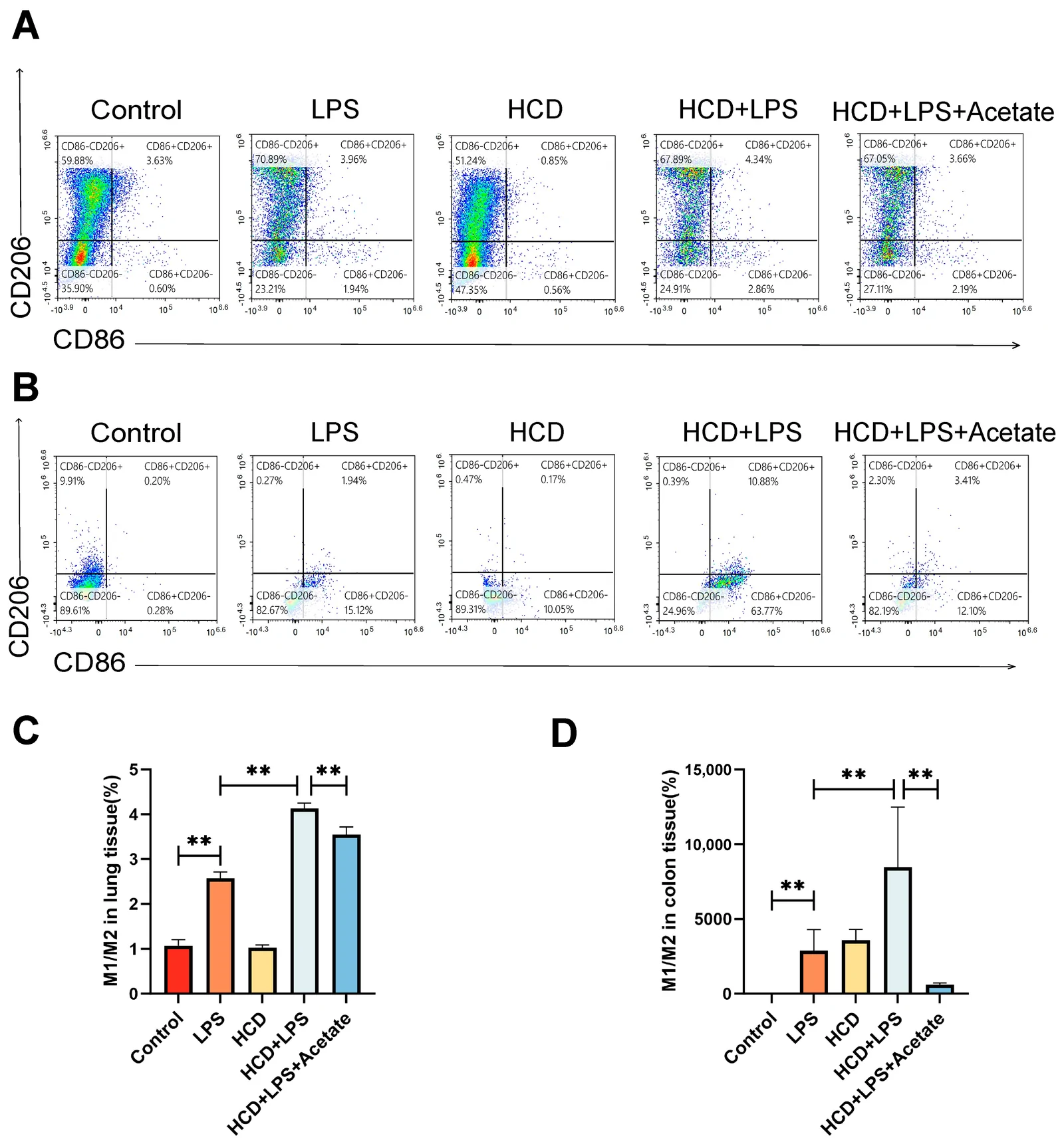
HCD exacerbates the M1/M2 macrophage imbalance in mice with LPS-induced pulmonary inflammation. (**A**) Flow cytometry analysis of CD86 and CD206 expression on lung macrophages; (**B**) Flow cytometry analysis of CD86 and CD206 expression on macrophages in the lamina propria of colon tissue; (**C**) The proportion of M1/M2 macrophages in the lung of each group; (**D**) The proportion of M1/M2 macrophages in the colon of each group. Results are presented as mean ± SD, with *n* = 6 mice per group for independent biological replicate experiments. ** *p* < 0.01.

**Figure 3:**
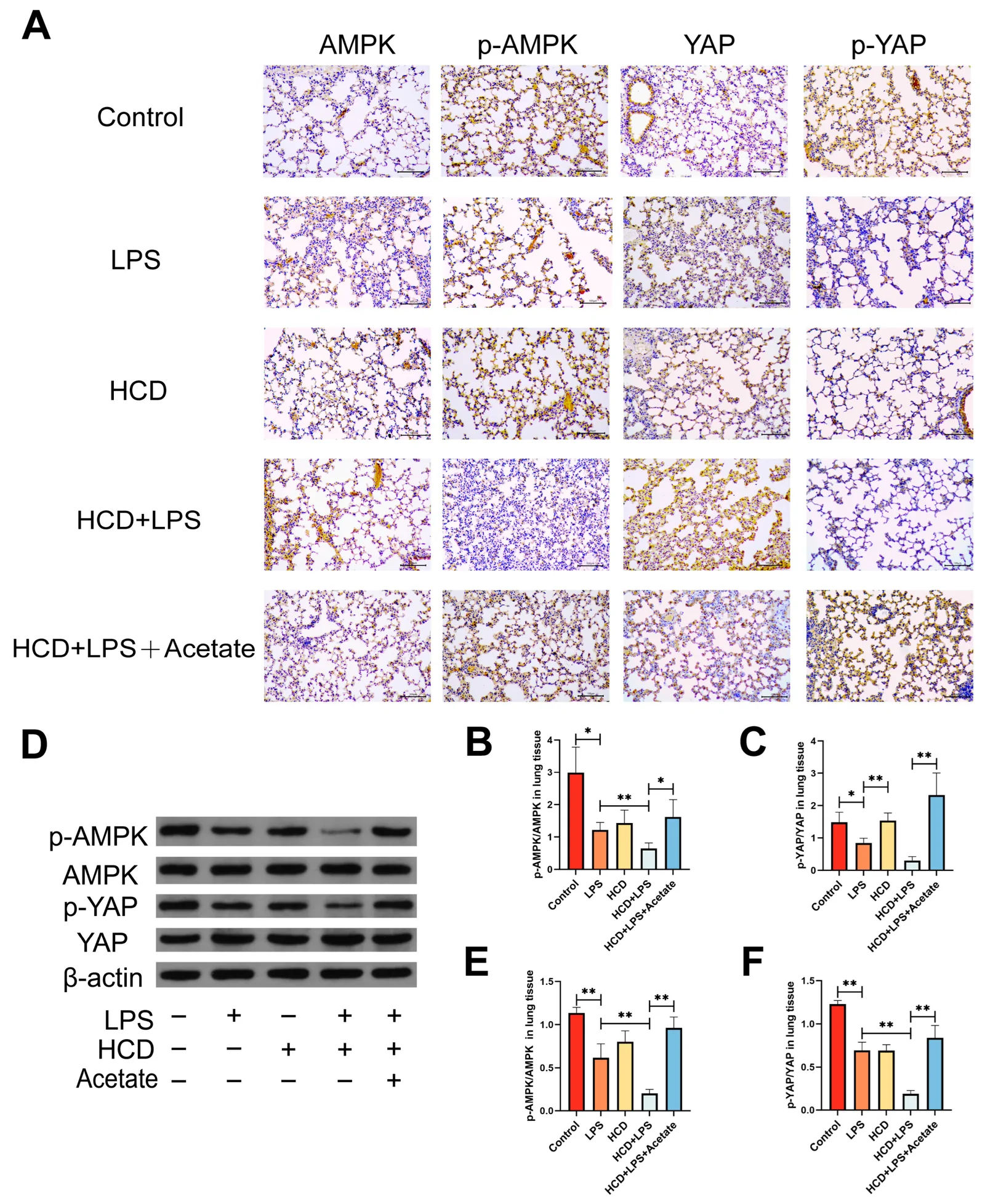
The mechanism by which HCD exacerbates M1/M2 macrophage polarization imbalance may be associated with the AMPK/YAP pathway. (**A**) Immunohistochemical detection of AMPK, YAP, *p*-AMPK, and *p*-YAP expression in lung and colon tissues of mice. (**B**) The ratio of *p*-AMPK/AMPK in lung tissues. Results are presented as mean ± SD with *n* = 6 mice per group for independent biological replicate experiments. (**C**) The ratio of *p*-YAP/YAP in lung tissues. Results are presented as mean ± SD with *n* = 6 mice per group for independent biological replicate experiments. (**D**) Representative Western blots of *p*-AMPK, AMPK, *p*-YAP and YAP. Results are presented as mean ± SD with *n* = 3 mice per group for independent biological replicate experiments. (**E**) Immunoblot quantification of AMPK phosphorylation in lung tissue. Results are presented as mean ± SD with *n* = 3 mice per group for independent biological replicate experiments. (**F**) Immunoblot quantification of YAP phosphorylation in lung tissue. Results are presented as mean ± SD with *n* = 3 mice per group for independent biological replicate experiments. ** *p* < 0.01; * *p* < 0.05.

**Figure 4:**
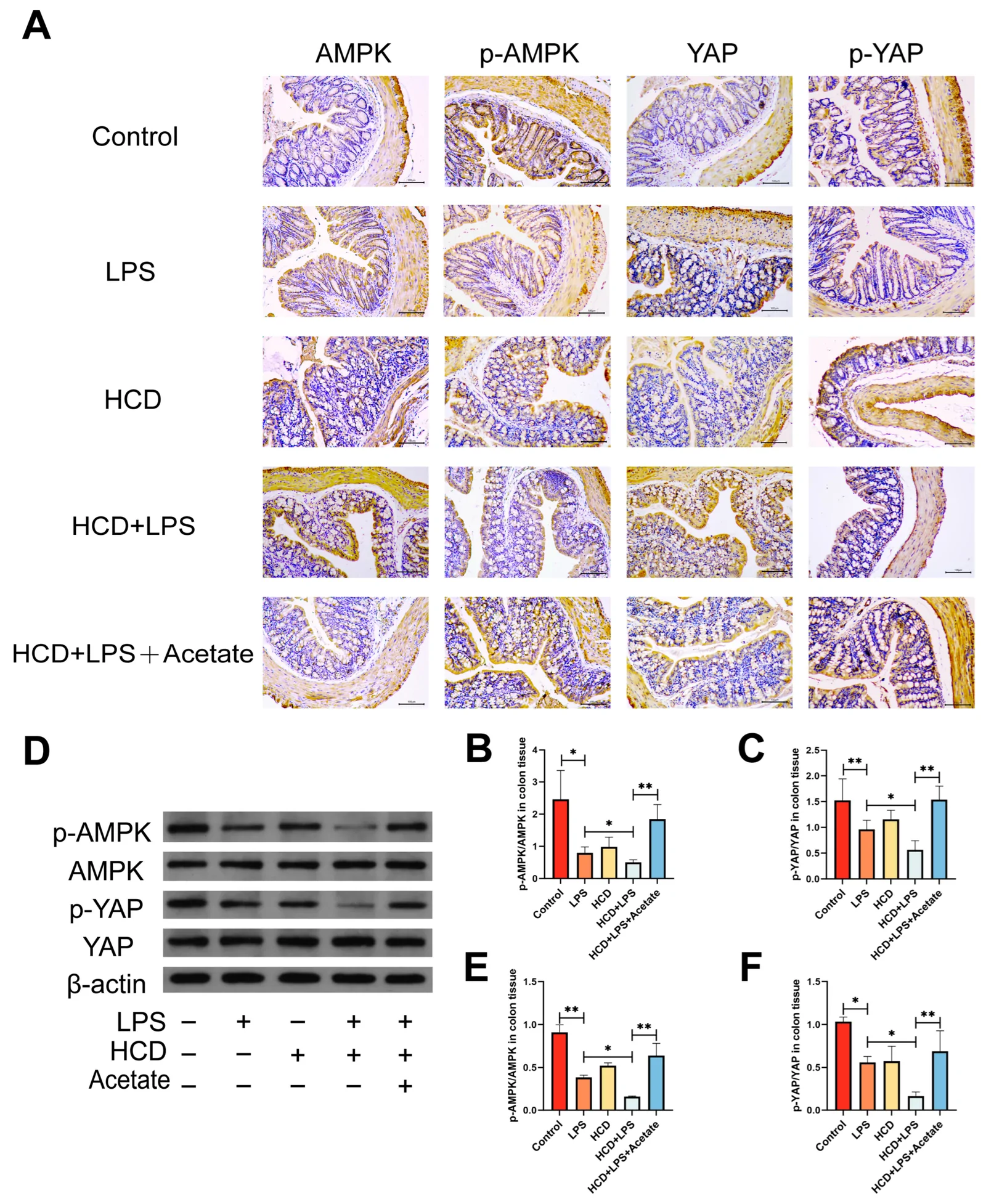
The HCD-induced exacerbation of M1/M2 macrophage polarization imbalance may be associated with the AMPK/YAP pathway. (**A**) Immunohistochemical detection of AMPK, YAP, *p*-AMPK, and *p*-YAP expression in colon tissues of mice. (**B**) The ratio of *p*-AMPK/AMPK in colon tissues. Results are presented as mean ± SD with *n* = 6 mice per group for independent biological replicate experiments. (**C**) The ratio of *p*-YAP/YAP in colon tissues. Results are presented as mean ± SD with *n* = 6 mice per group for independent biological replicate experiments. (**D**) Representative Western blots of *p*-AMPK, AMPK, *p*-YAP and YAP. Results are presented as mean ± SD with *n* = 3 mice per group for independent biological replicate experiments. (**E**) Immunoblot quantification of AMPK phosphorylation in colon tissue. Results are presented as mean ± SD with *n* = 3 mice per group for independent biological replicate experiments. (**F**) Immunoblot quantification of YAP phosphorylation in colon tissue. Results are presented as mean ± SD with *n* = 3 mice per group for independent biological replicate experiments. ** *p* < 0.01; * *p* < 0.05.

**Figure 5:**
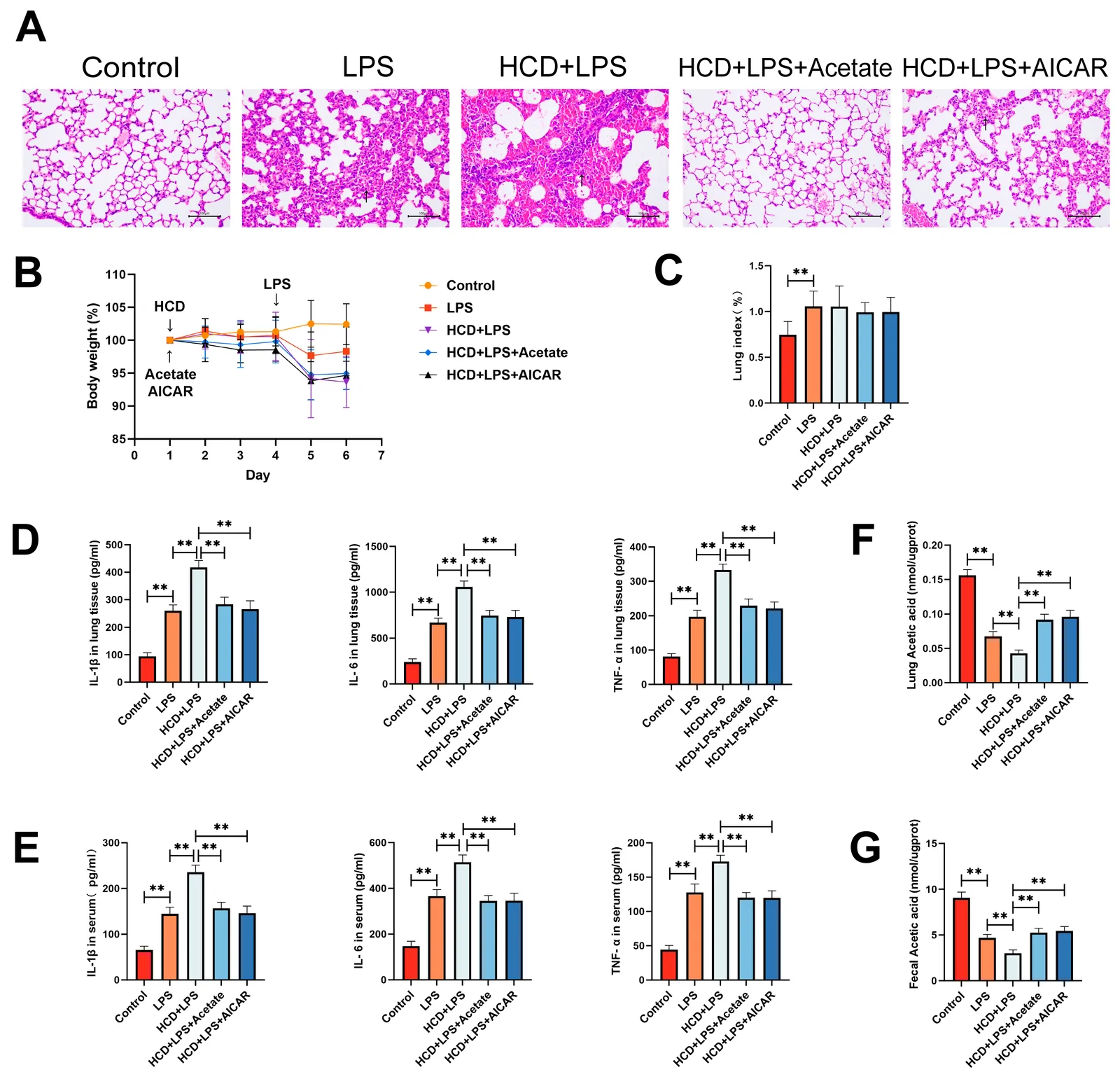
Supplementation with acetate and activation of AMPK were associated with mitigation of LPS- and HCD-induced pulmonary inflammation. (**A**) Lung H&E staining results (200×). The black arrow indicates areas of inflammatory cell infiltration and alveolar wall thickening. (**B**) Relative body weight changes during the experimental period. Results are presented as mean ± SD, with *n* = 10 mice per group for independent biological replicate experiments. (**C**) Lung index. Results are presented as mean ± SD, with *n* = 10 mice per group for independent biological replicate experiments. (**D**,**E**) ELISA determination of pro-inflammatory cytokines (IL-6, IL-1β, TNF-α) in lung and serum samples from each group; (**F**,**G**) Acetate content in lung and fecal samples from each group, measured by an acetate assay kit. Results are presented as mean ± SD with *n* = 6 mice per group for independent biological replicate experiments. ** *p* < 0.01.

**Figure 6:**
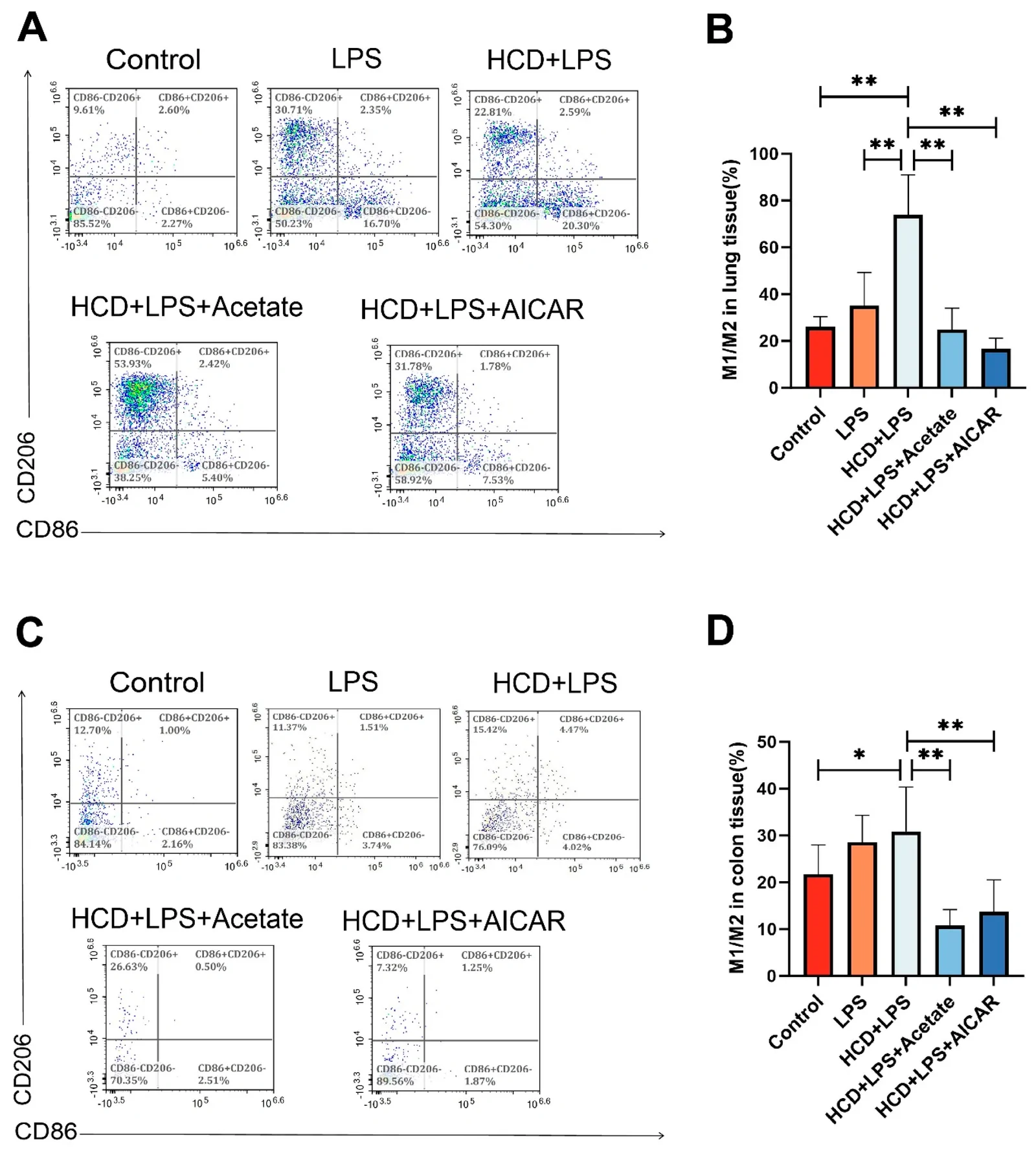
HCD exacerbates the M1/M2 macrophage imbalance in mice with LPS-induced pulmonary inflammation. (**A**) Flow cytometry analysis of CD86 and CD206 expression on lung macrophages; (**B**) The proportion of M1/M2 macrophages in the lung of each group; (**C**) Flow cytometry analysis of CD86 and CD206 expression on macrophages in the lamina propria of colon tissue; (**D**) The proportion of M1/M2 macrophages in the colon of each group. Results are expressed as mean ± SD with *n* = 6 mice per group for independent biological replicate experiments. ** *p* < 0.01; * *p* < 0.05.

**Figure 7:**
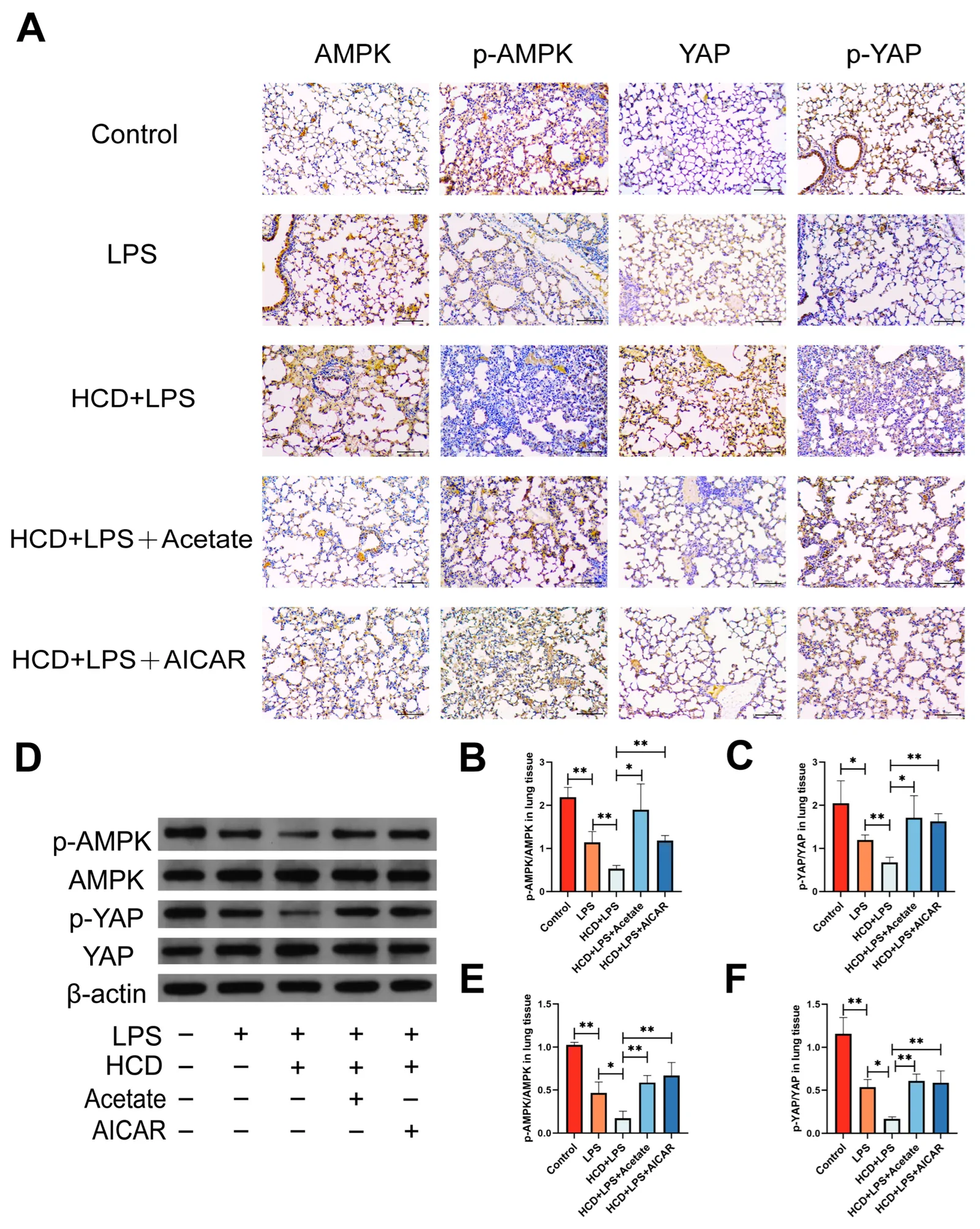
Acetate supplementation was consistently associated with alterations in the AMPK/YAP pathway and with increased AMPK activation in lung tissue. (**A**) Immunohistochemical detection of AMPK, YAP, *p*-AMPK, and *p*-YAP in lung tissues of mice. (**B**) The *p*-AMPK/AMPK ratio by IHC in lung tissues. Results are presented as mean ± SD with *n* = 6 mice per group for independent biological replicate experiments. (**C**) The *p*-YAP/YAP ratio by IHC in lung tissues. Results are presented as mean ± SD with *n* = 6 mice per group for independent biological replicate experiments. (**D**) Representative Western blots of *p*-AMPK, AMPK, *p*-YAP and YAP. Results are presented as mean ± SD with *n* = 3 mice per group for independent biological replicate experiments. (**E**) Immunoblot quantification of AMPK phosphorylation in lung tissue. Results are presented as mean ± SD with *n* = 3 mice per group for independent biological replicate experiments. (**F**) Immunoblot quantification of YAP phosphorylation in lung tissue. Results are presented as mean ± SD with *n* = 3 mice per group for independent biological replicate experiments. ** *p* < 0.01; * *p* < 0.05.

**Figure 8:**
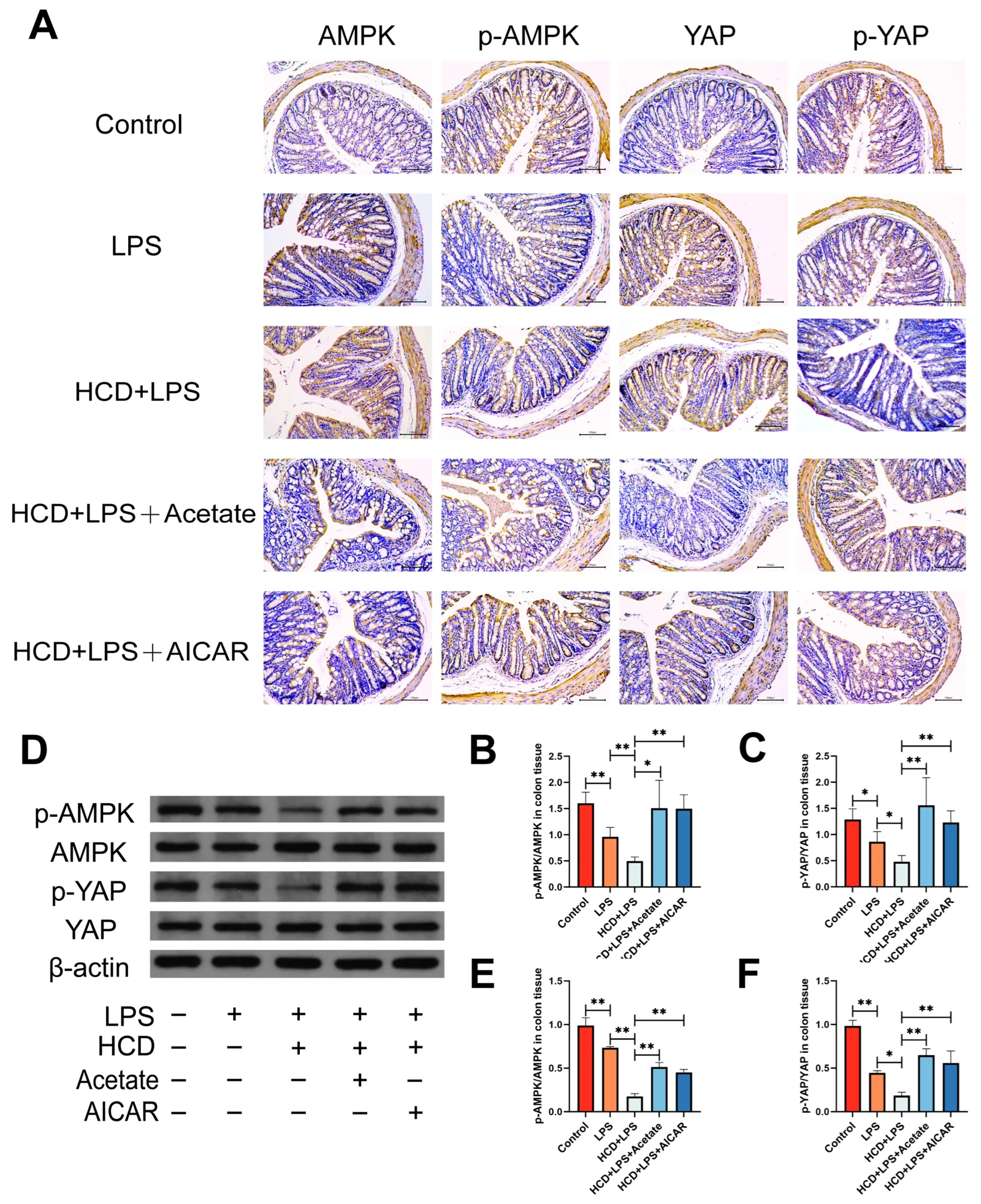
Acetate supplementation was consistently associated with changes in the AMPK/YAP pathway and with AMPK activation in colon tissue. (**A**) Immunohistochemical detection of AMPK, YAP, *p*-AMPK, and *p*-YAP in colon tissues of mice. (**B**) The *p*-AMPK/AMPK ratio by IHC in colon tissues. Results are presented as mean ± SD with *n* = 6 mice per group for independent biological replicate experiments. (**C**) The *p*-YAP/YAP IHC ratio in colon tissues. Results are presented as mean ± SD with *n* = 6 mice per group for independent biological replicate experiments. (**D**) Representative Western blots of *p*-AMPK, AMPK, *p*-YAP and YAP. Results are presented as mean ± SD with *n* = 3 mice per group for independent biological replicate experiments. (**E**) Immunoblot quantification of AMPK phosphorylation in colon tissue. Results are presented as mean ± SD with *n* = 3 mice per group for independent biological replicate experiments. (**F**) Immunoblot quantification of YAP phosphorylation in colon tissue. Results are presented as mean ± SD with *n* = 3 mice per group for independent biological replicate experiments. ** *p* < 0.01; * *p* < 0.05.

**Figure 9:**
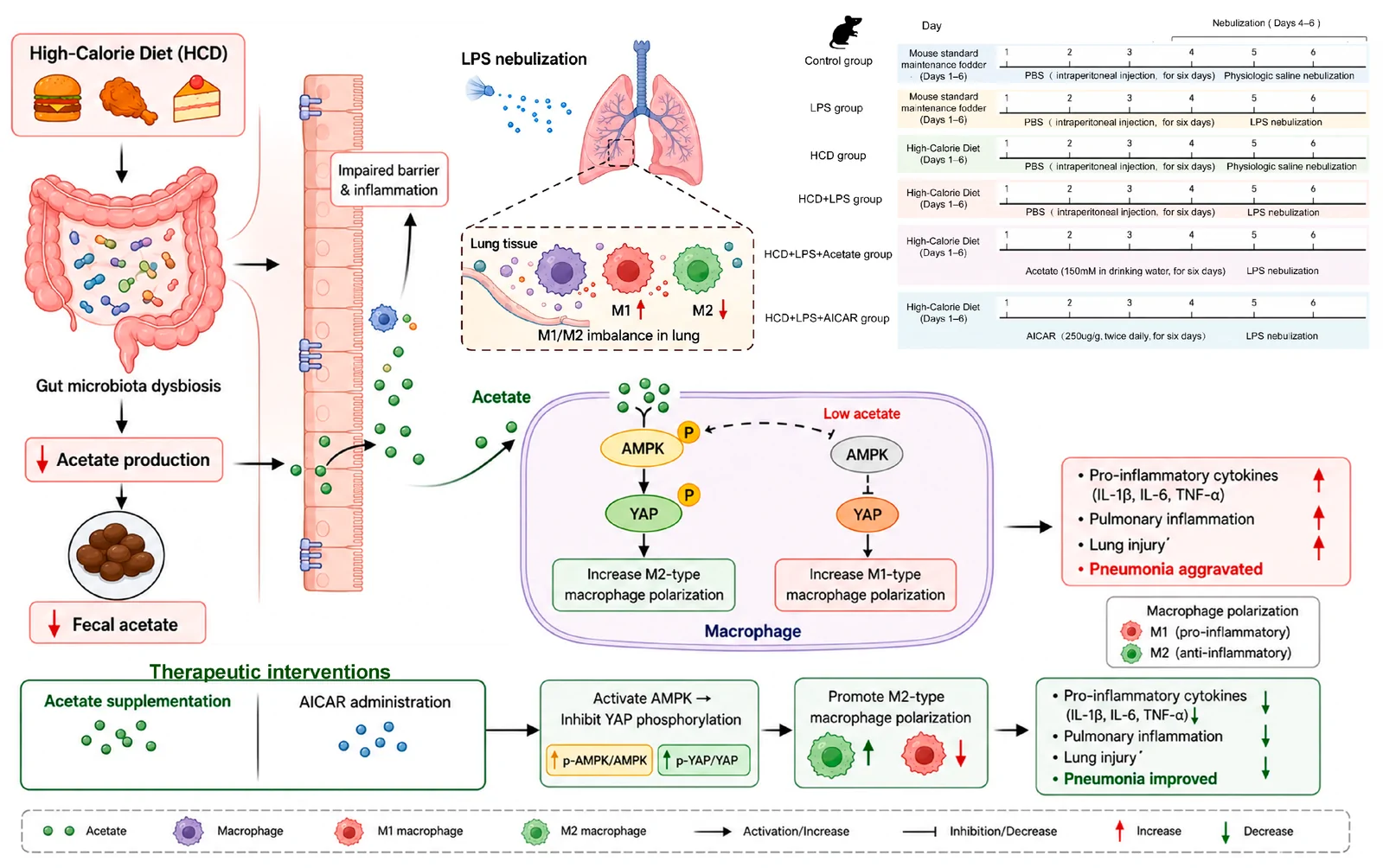
Schematic diagram of the mechanism by which an HCD exacerbates pneumonia.

**Table 1:** Experiment 1 protocol.

Group	*n*	Feed (1–6 Days)	Atomization (4–6 Days)
Control	10	Mouse standard maintenance fodder	Physiologic saline
HCD	10	High-calorie diet	Physiologic saline
LPS	10	Mouse standard maintenance fodder	LPS solution
HCD + LPS	10	High-calorie diet	LPS solution
HCD + LPS + Acetate	10	High-calorie diet	LPS solution

**Table 2:** Experiment 2 protocol.

Group	*n*	Feed (1–6 Days)	Atomization (4–6 Days)
Control	10	Mouse standard maintenance fodder	Physiologic saline
LPS	10	Mouse standard maintenance fodder	LPS solution
HCD + LPS	10	High-calorie diet	LPS solution
HCD + LPS + Acetate	10	High-calorie diet	LPS solution
HCD + LPS + AICAR	10	High-calorie diet	LPS solution

## Data Availability

Data and materials may be made available upon written request to the corresponding author.

## Supplementary Materials

The following supporting information can be downloaded at: https://www.mdpi.com/article/10.3390/nu18193196/s1. Figure S1: Colon H&E staining results (200×).; Figure S2: Flow cytometry gating strategy.; Figure S3: Correlation analysis among AMPK/YAP pathway activity, M1/M2 macrophage cell ratio, and pro-inflammatory cytokine levels in lung and colon tissues. **(A)** Correlation analysis between the p-AMPK/AMPK ratio and pro-inflammatory cytokine levels in lung tissue. (**B**) Correlation analysis between the p-YAP/YAP ratio and pro-inflammatory cytokine levels in lung tissue. (**C**) Correlation analysis between the p-AMPK/AMPK ratio and pro-inflammatory cytokine levels in colon tissue. (**D**) Correlation analysis between the p-YAP/YAP ratio and pro-inflammatory cytokine levels in colon tissue. (**E**) Correlation analysis among the p-AMPK/AMPK ratio, M1/M2 ratio in lung tissue and p-YAP/YAP ratio in lung tissue. (**F**) Correlation analysis between the p-AMPK/AMPK ratio in lung tissue and M1/M2 ratio in colon tissue. (**G**) Correlation analysis between the p-YAP/YAP ratio and M1/M2 ratio in lung tissue. (**H**) Correlation analysis between the p-YAP/YAP ratio in lung tissue and M1/M2 ratio in colon tissue. (**I**) Correlation analysis among the p-AMPK/AMPK ratio in colon tissue, M1/M2 ratio in lung tissue and p-YAP/YAP ratio in colon tissue. (**J**) Correlation analysis between the p-YAP/YAP ratio in colon tissue and M1/M2 ratio in lung tissue. (**K**) Correlation analysis between the p-YAP/YAP ratio and M1/M2 ratio in colon tissue. (**L**) Correlation analysis between the p-AMPK/AMPK ratio and M1/M2 ratio in colon tissue. Results are presented as mean ± SD, with n = 6 mice per group; Figure S4: Colon H&E staining results (200×); Figure S5: Flow cytometry gating strategy.

## Abbreviations

The following abbreviations are used in this manuscript:

SCFAs	Short-chain fatty acids
LPS	Lipopolysaccharide
AMPK	Adenosine 5′-monophosphate (AMP)-activated protein kinase
YAP	Yes-associated protein
AICAR	5-Aminoimidazole-4-carboxamide ribonucleoside
IL-1β	Interleukin-1 beta
IL-6	Interleukin-6
TNF-α	Tumor necrosis factor-alpha
GPR41	G-protein-coupled receptor 41
GPR43	G-protein-coupled receptor 43
HDAC	Histone deacetylases
NF-κB	Nuclear factor kappa-B
PPARγ	Peroxisome proliferator-activated receptor γ
