# Supplementary material for: High-Calorie Diet Aggravates Lipopolysaccharide-Induced Pulmonary Inflammation in Juvenile Mice: Association of Acetate-Mediated Macrophage Polarization with AMPK/YAP Signaling
Source: Nutrients. 2026 Sep 27;18(19):3196. doi: 10.3390/nu18193196 (PMC13667027; doi:10.3390/nu18193196)
Supplement: Supplementary file 1 [file nutrients-18-03196-s001.zip › Supplementary file.pdf]

Supplementary file

1 Experiment 1: Colon H&E staining results

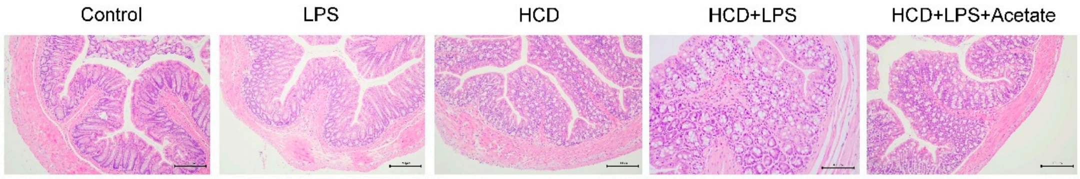

Figure S1 Colon H&E staining results (200×).

2 Experiment 1: Flow Cytometric Gating Strategy

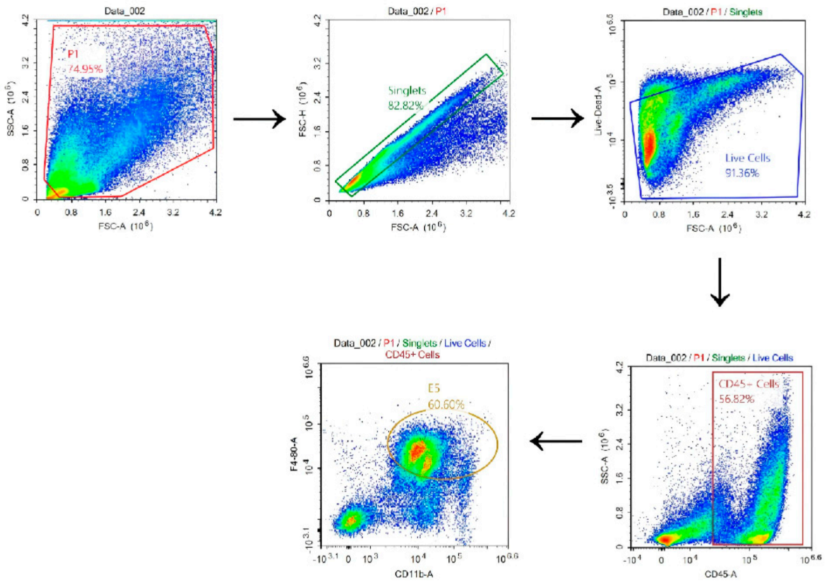

Figure S2 Flow cytometry gating strategy.

3 Experiment 1: Correlation analysis among AMPK/YAP pathway activity, M1/M2 macrophage cell ratio, and pro-inflammatory cytokine levels in lung and colon tissues.

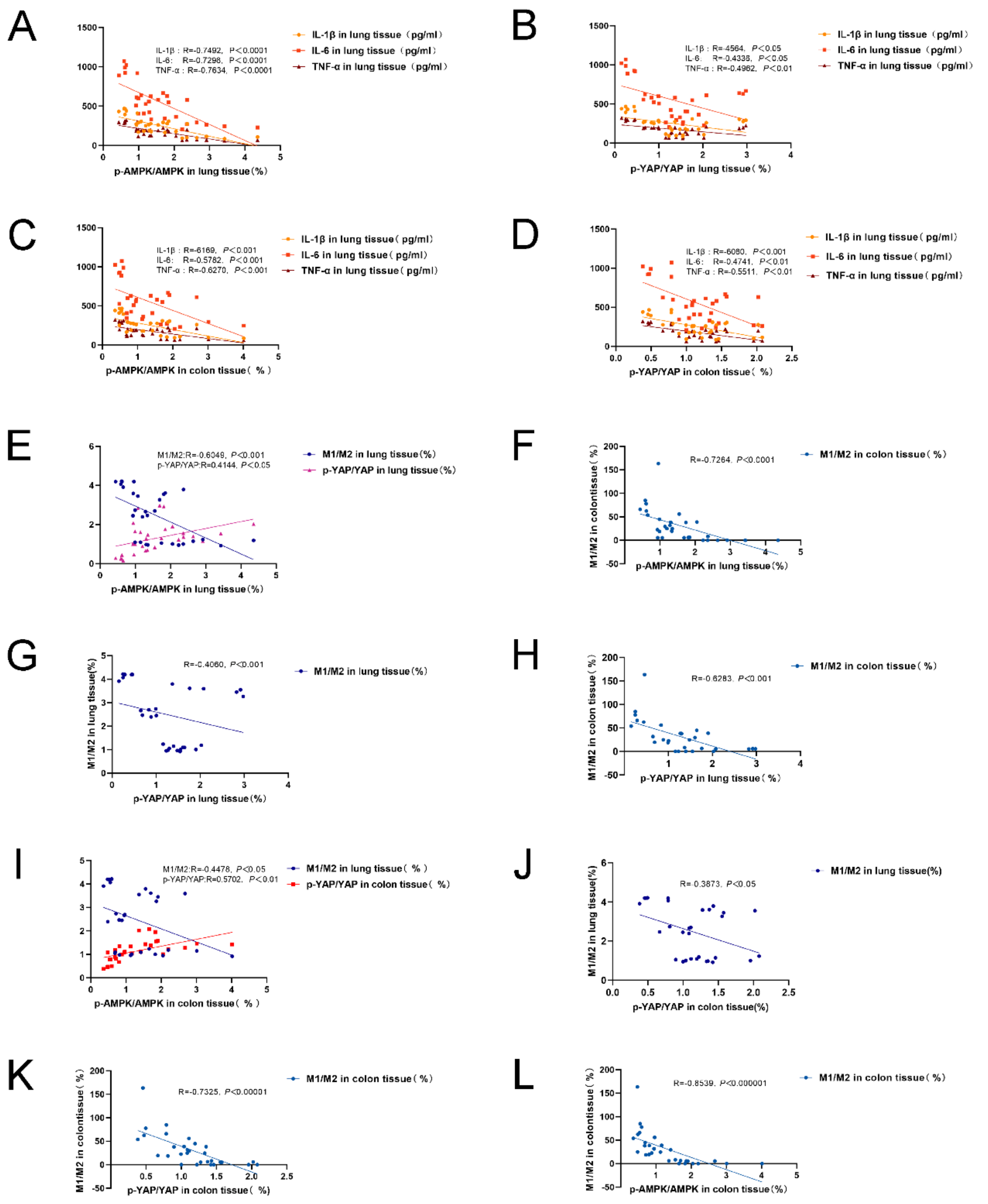

Figure S3 Correlation analysis among AMPK/YAP pathway activity, M1/M2 macrophage cell ratio, and pro-inflammatory cytokine levels in lung and colon tissues. **(A)**Correlation analysis between the p-AMPK/AMPK ratio and pro-inflammatory cytokine levels in lung tissue. (**B**) Correlation analysis between the p-YAP/YAP ratio and pro-inflammatory cytokine levels in lung tissue. (**C**) Correlation analysis between the p-AMPK/AMPK ratio and pro-inflammatory cytokine levels in colon tissue. (**D**) Correlation analysis between the p-YAP/YAP ratio and pro-inflammatory cytokine levels in colon tissue. (**E**) Correlation analysis among the p-AMPK/AMPK ratio, M1/M2 ratio in lung tissue and p-YAP/YAP ratio in lung tissue. (**F**) Correlation analysis between the p-AMPK/AMPK ratio in lung tissue and M1/M2 ratio in colon tissue. (**G**) Correlation analysis between the p-YAP/YAP ratio and M1/M2 ratio in lung tissue. (**H**)

Correlation analysis between the p-YAP/YAP ratio in lung tissue and M1/M2 ratio in colon tissue. (**I**) Correlation analysis among the p-AMPK/AMPK ratio in colon tissue, M1/M2 ratio in lung tissue and p-YAP/YAP ratio in colon tissue. (**J**) Correlation analysis between the p-YAP/YAP ratio in colon tissue and M1/M2 ratio in lung tissue. (**K**) Correlation analysis between the p-YAP/YAP ratio and M1/M2 ratio in colon tissue. (**L**) Correlation analysis between the p-AMPK/AMPK ratio and M1/M2 ratio in colon tissue. Results are presented as mean ± SD, with n = 6 mice per group.

4 Experiment 2: Colon H&E staining results

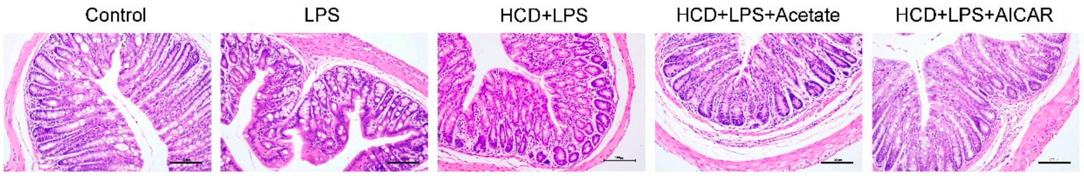

Figure S4 Colon H&E staining results (200×).

5 Experiment 2: Flow Cytometric Gating Strategy

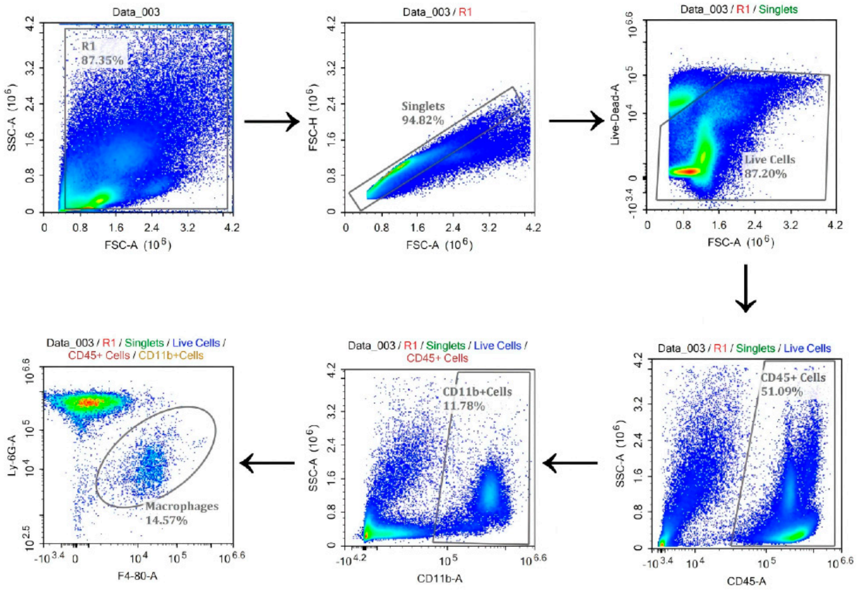

Figure S5 Flow cytometry gating strategy.
